# Nitrogen Accumulation in Oyster (*Crassostrea gigas*) Slurry Exposed to Virucidal Cold Atmospheric Plasma Treatment

**DOI:** 10.3390/life11121333

**Published:** 2021-12-02

**Authors:** Isabella Csadek, Peter Paulsen, Pia Weidinger, Kathrine H. Bak, Susanne Bauer, Brigitte Pilz, Norbert Nowotny, Frans J. M. Smulders

**Affiliations:** 1 Unit of Food Hygiene and Technology, Food Technology and Veterinary Public Health, Institute of Food Safety, University of Veterinary Medicine Vienna, Veterinärpl. 1, 1210 Vienna, Austria; peter.paulsen@vetmeduni.ac.at (P.P.); kathrine.bak@vetmeduni.ac.at (K.H.B.); susanne.bauer@vetmeduni.ac.at (S.B.); brigitte.pilz@vetmeduni.ac.at (B.P.); frans.smulders@vetmeduni.ac.at (F.J.M.S.); 2 Viral Zoonoses, Emerging and Vector-Borne Infections Group, Institute of Virology, University of Veterinary Medicine Vienna, Veterinärpl. 1, 1210 Vienna, Austria; pia.weidinger@vetmeduni.ac.at (P.W.); norbert.nowotny@vetmeduni.ac.at (N.N.); 3 Department of Basic Medical Sciences, College of Medicine, Mohammed Bin Rashid University of Medicine and Health Sciences, Dubai Healthcare City, Dubai, United Arab Emirates

**Keywords:** herpesvirus, coronavirus, cold atmospheric plasma, oysters, mussels, nitrogen accumulation

## Abstract

Viral contamination of edible bivalves is a major food safety issue. We studied the virucidal effect of a cold atmospheric plasma (CAP) source on two virologically different surrogate viruses [a double-stranded DNA virus (Equid alphaherpesvirus 1, EHV-1), and a single-stranded RNA virus (Bovine coronavirus, BCoV)] suspended in Dulbecco’s Modified Eagle’s Medium (DMEM). A 15 min exposure effectuated a statistically significant immediate reduction in intact BCoV viruses by 2.8 (ozone-dominated plasma, “low power”) or 2.3 log cycles (nitrate-dominated, “high power”) of the initial viral load. The immediate effect of CAP on EHV-1 was less pronounced, with “low power” CAP yielding a 1.4 and “high power” a 1.0 log reduction. We observed a decline in glucose contents in DMEM, which was most probably caused by a Maillard reaction with the amino acids in DMEM. With respect to the application of the virucidal CAP treatment in oyster production, we investigated whether salt water could be sanitized. CAP treatment entailed a significant decline in pH, below the limits acceptable for holding oysters. In oyster slurry (a surrogate for live oysters), CAP exposure resulted in an increase in total nitrogen, and, to a lower extent, in nitrate and nitrite; this was most probably caused by absorption of nitrate from the plasma gas cloud. We could not observe a change in colour, indicative for binding of NO_x_ to haemocyanin, although this would be a reasonable assumption. Further studies are necessary to explore in which form this additional nitrogen is deposited in oyster flesh.

## 1. Introduction

A number of studies demonstrate the ability of cold atmospheric plasma (CAP) devices to generate reactive oxygen (ROS) and reactive nitrogen species (RNS) in a way that the viability and integrity of bacteria and viruses on food surfaces [1,2,3,4,5,6,7,8,9] and food packaging [10] exposed to such gas species can be substantially impaired. Regarding proteinaceous food, such as meat and seafood, most studies report minor or no changes in sensory quality traits, such as colour and pH [11,12,13,14]. Exposure of haem pigments to RNS can lead to the formation of nitrosomyoglobin (MbNO), showing a pink (cured) colour, whereas effects of ROS on haem pigments are less explored [15,16]. Currently, curing of meat is under heavy debate [17,18,19] and in non-meat products, nitrate is mainly regarded as a contaminant [20,21]. This applies also to those aquatic species which do not only absorb oxygen from the surrounding water, but also filtrate it to retain smaller organisms as food (“filter feeders”, [22]).

Seafood, in particular mussels and Ostreidae, has been implicated in foodborne bacterial and viral poisoning [23,24,25,26,27,28,29,30,31,32], and the control of biological hazards in these food items is a challenging task in food safety assurance [33,34,35,36,37]. This is especially as most mussel species are traded alive and some are consumed raw, which makes heat treatment [38] as a control measure impossible. Recently, it has been demonstrated that a jet-type CAP device can reduce surrogate foodborne viruses in an oyster (*Crassostrea gigas*) slurry without compromising colour and pH [39], and it can be speculated that CAP treatment of the surrounding water [40] could effectuate disinfection of live oysters.

Unlike meat, the flesh of seafood carries haemocyanin as the electron acceptor [41]. Haemocyanin is colourless in its deoxygenated form and blue when oxygenated [42]. Verplaetse et al. [43] cite previous studies finding that formation of nitrosylhaemocyanin results in a green colour. Thus, any colour changes in seafood induced by CAP (blue or green) are expected to be different from the CAP-induced colour changes in meat [15].

The aim of our study was, firstly, to define settings for a dielectric barrier discharge (DBD)-type CAP generator [12,44,45,46,47,48,49,50,51] to achieve a reduction in two different viruses suspended in cell culture medium. Secondly, to use these settings for the treatment of oyster slurry, to study whether the exposure to ROS or RNS would result in changes in its chemical composition, in particular nitrogen contents, as such changes would raise the question of whether CAP-treated food has to be considered as “novel food” according to EU legislation [52]. Finally, to investigate if such treatment delivering RNS species would considerably raise the NO_x_ levels in oysters.

## 2. Materials and Methods

### 2.1. Plasma-Generating Device

We used a DBD system as the CAP-generating device which was developed at the University of Liverpool and has a construction similar to that used in the study by Ni et al. [53]. The flat electrodes were separated by a dielectric quartz plate. A grounded metal mesh electrode (approx.8 cm × 8 cm) was directed towards the sample. A sinusoidal high-voltage signal (settings see Table 1) was applied to generate plasma from the ambient air. By increasing the voltage, the plasma composition shifts from an O_3_ (ROS) to a nitrate/NO_x_ (RNS)-dominated gas [12].

### 2.2. Assessment of Virucidal Activity in Dulbecco’s Modified Eagle’s Medium

To assess the virucidal activity of CAP, the total amount of viral nucleic acid in two different virus isolates suspended in Dulbecco’s Modified Eagle’s Medium (DMEM, Gibco, Dublin, Ireland) was quantified by real-time (reverse transcription) polymerase chain reaction [(RTqPCR)] before and after CAP exposure. In addition, the ozone content in DMEM was determined.

#### 2.2.1. Virus Isolates

Both virus isolates—i.e., Equid alphaherpesvirus 1 (EHV-1) strain Kentucky D (7th passage at the Institute of Virology, University of Veterinary Medicine Vienna), and Bovine coronavirus (BCoV, a Betacoronavirus 1) strain 15317/82 in its third passage—have been stored in DMEM at −80 °C since their isolation from cell culture in 2014 and 2020, respectively.

#### 2.2.2. Virus Detection

In order to destroy any free DNA or RNA molecules that emerged during the treatment with CAP, and to ensure that only intact virus particles were detected, each EHV-1 sample was subjected to DNase treatment, and each BCoV sample to RNase treatment after their exposure to CAP. For this, 120 µL of each EHV-1 sample were mixed with 15 µL reaction buffer, 1.5 µL DNase I, and 13.5 µL nuclease-free H_2_O (Kit M0303, New England Biolabs, Ipswich, MA, USA), and incubated for 10 min at 37 °C, while 120 µL of each BCoV sample was mixed with 20 µL RNase A (1 µg/µL; AppliChem, Darmstadt, Germany) and incubated for 1 h at 37 °C.

Thereafter, the nucleic acids of each sample were extracted using QIAamp Viral RNA Mini Kit on a QIAcube automatic device (both Qiagen, Hilden, Germany). After the extraction, EHV-1 samples were subjected to an EHV-1-specific quantitative PCR (qPCR) using Luna Universal Probe qPCR Master Mix (New England Biolabs, Ipswich, MA, USA) and the following primers: EHV1-F 5′-ATCTGGCCGGGCTTCAAC-3′ and EHV1-R 5′-GGTCACCCACCTCGAACGT-3′ together with the probe EHV1-P 5′-FAM-ATCCGTCRACTACTCG-TAMRA-3′, amplifying a 54 bp-long sequence within the open reading frame (ORF) 30 of EHV-1 under the following conditions: 95 °C, 1 min; and 45 cycles 95 °C, 15 s and 60 °C, 30 s. BCoV samples were screened via RT-qPCR using qScript XLT One Step RT-qPCR ToughMix (QuantaBio, Beverly, MA, USA) and the following primers: βCoV-F 5′-ACGTGGTGTTCCTGTTGTTATAGG-3′ and βCoV-R 5′-AACATCTTTAATAAGGCGACGTAACAT-3′, together with the probe βCoV-P 5′-FAM-CCACTAAGTTTTATGGCGGCTGGGATG-TAMRA-3′, amplifying an approximately 100 bp-long sequence within the RNA polymerase (pol) gene of many betacoronaviruses under the following conditions: 50 °C, 15 min; 95 °C, 2 min; and 45 cycles 95 °C, 15 s and 60 °C, 30 s. All (RT-)qPCRs were performed either on Applied Biosystems 7500 (Waltham, MA, USA) or Rotor-Gene Q (Qiagen, Hilden, Germany) real-time PCR systems. The difference between the cycle threshold (Ct) values of CAP-exposed and non-exposed samples (controls) was used to determine the reduction in the viral load after CAP treatment.

#### 2.2.3. Determination of Ozone in DMEM

To evaluate the functionality of the device and quantify the ozone generation, we exposed DMEM to CAP and then quantified the generated ozone with colorimetric methods (MColortest Ozone Test, Merck, Darmstadt, Germany and Ozone Test, Macherey-Nagel, Düren, Germany).

#### 2.2.4. Experimental Setup for Viral Reduction Using CAP

**Figure 1:**
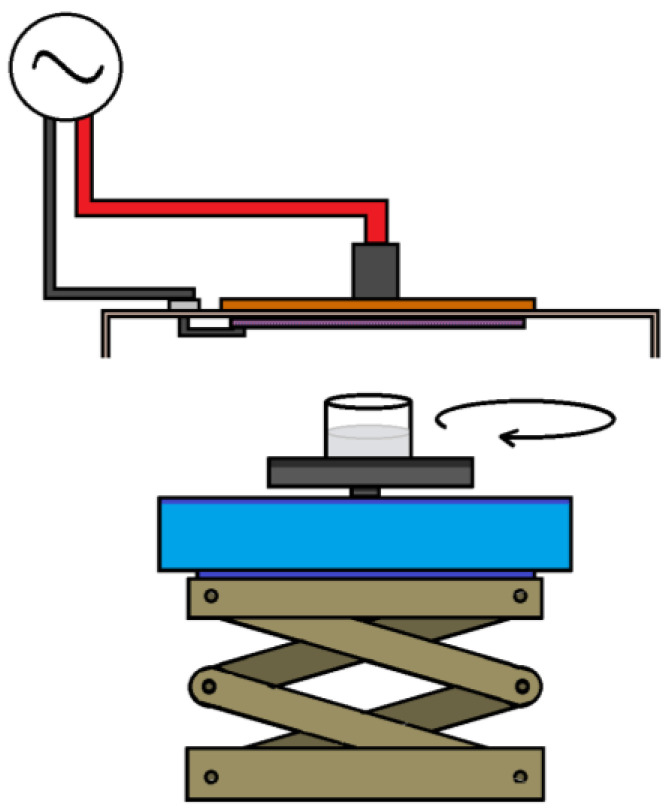
Experimental setup; the plasma source extends over a well containing the sample. The well is placed on an orbital shaker to ensure uniform exposure of the sample to CAP; the shaker is placed on a height-adjustable stand.

Viral suspensions were brought to room temperature before CAP exposure. Then, 100 μL of each virus isolate (EHV-1; BCoV) were mixed with 500 μL DMEM and dispensed in one well of a 12-well plate (23 mm in diameter). The plate was placed on an orbital shaker (Minishaker MS1, IKA, Staufen, Germany) (at 800 rpm) and a plasma source was adjusted above to achieve a distance of 10 mm from the electrode mesh to the surface of the liquid (Figure 1). The wells were exposed to “high” or “low power” CAP, for 5, 10, or 15 min each.

After the treatment, the samples were divided into two fractions and pipetted into polyethylene vessels (Eppendorf Tubes 3810X, Eppendorf, Hamburg, Germany): one was stored for 7 days at 4–8 °C, while the other one was immediately frozen at −80 °C. Both fractions were then further processed and examined by (RT-)qPCR as described above.

### 2.3. Effect of CAP on Liquid Matrices

The array of liquid matrices and performed tests is described below. All matrices were tested before and after CAP exposure (“high” or “low power” for 5, 10, or 15 min) with the same volume and device configuration as described above.

- Tap water (unbuffered control) was tested for pH and temperature.
- DMEM (nutrient-rich, buffered medium) was tested for pH, temperature, and glucose content (D-Glucose/D-Fructose Enzymatic test Kit no. 10139106035, R-Biopharm, Darmstadt, Germany).
- Salt water (the typical oyster-holding medium) was prepared by adding 38 g sea salt (Premium Reef Salt, ARKA Biotechnologie, Röthenbach, Germany) to 1 L distilled water. The average pH was 8.1, with a density of 1.022 g/cm^3^ at 25 °C. Typical mineral contents were potassium (430 mg/L) and magnesium (1300 mg/L). Nitrates and phosphates were not detectable. Salt water was tested for pH and temperature.

### 2.4. Effect of CAP on Oyster Slurry

Oyster slurry was obtained by grinding frozen oysters (Ostreidae) obtained from a local grocery store. Temperature, pH, colour, and nitrogen content were determined before and after CAP exposure. In addition, we determined the contents of NO_x_ in oyster slurry mixed with sodium nitrite and potassium nitrate (Roth, Darmstadt, Germany). Colour measurement was performed by reflectance spectrometry (Nix QC Scan, Nix Sensor Ltd., Hamilton, ON, Canada) to record L* (lightness), a* (redness), and b* (yellowness) values, as well as ΔE* (total colour difference). Total nitrogen was determined according to the Kjeldahl method [54]. Determination of nitrate and nitrite in the oyster slurry was performed by high-performance liquid chromatography (HPLC; Alliance 2695, Waters, Milford, MA, USA) with detection by a photodiode array detector (PDA) at 205 nm (PDA 996, Waters, Milford, MA, USA), according to a method adapted from Schmidt and Schwedt [55] with minor modifications.

Approximately 2.5 g of the oyster slurry was weighed into a 100 mL beaker and homogenised with 20 mL HPLC-grade water. After heating for 10 min in a water bath (at 100 °C), the mixture was cooled and subsequently filtered through a folded filter paper (MN 615 ¼, Macherey-Nagel, Düren, Germany). The filtrate was then transferred to HPLC vials via a 0.2 µm membrane filter (Chromafil Xtra PET-20/25, Macherey-Nagel, Düren, Germany), and this final filtrate was used for the HPLC analysis. A sample size of 25 µL was separated on a Spherisorb S5 ODS2 250 × 4.6 mm column (Waters, Milford, MA, USA) at 20 °C. The mobile phase ran isocratically at a flow rate of 0.5 mL/min with 95% mobile phase A [buffer of dipotassium hydrogen phosphate (10 g/L) adjusted to pH 3 with ortho-phosphoric acid] and 5% mobile phase B (acetonitrile). For the quantification, standard solutions were prepared from KNO_3_ and NaNO_2_ and a calibration curve was created for both nitrate and nitrite. Detection was performed via a PDA detector at 205 nm. Weight loss during CAP exposure was determined by weighing the samples before and after CAP treatment.

### 2.5. Statistical Processing of Results

Data on temperature and pH were analysed by one-way ANOVA, with the duration of CAP exposure as the independent factor. Likewise, Ct values were analysed with power setting, nitrogen contents, and the duration of exposure as independent factors. *Tukey´s post-hoc test* was used to discriminate between means. The concentration of glucose in DMEM, before and after 15 min of CAP exposure, was analysed by a paired *t*-test. Statistical significance was established at *p* < 0.05.

## 3. Results and Discussion

### 3.1. Setup for Generating CAP

Dielectric barrier discharge (DBD) is a widely used design to generate plasma [56] which allows simultaneous exposure of larger surface areas than a plasma “jet” or “torch”. The actual composition of the plasma that contacts the sample will not only depend on the gas and voltage used for generating the plasma, but also on the distance of the electrode to the sample, since a larger distance will select for long-lived molecule and ion species. Together with varying time of exposure, a plethora of different experimental settings can be applied, and this is in fact observed when reviewing scientific papers [56]. We thus used device settings (voltage, current, and distance electrode-to-sample) that had demonstrated antibacterial effects without compromising the sample matrix [12], and varied only the duration of exposure. This is also a limitation of the study, since other electrical settings might have generated a higher density in reactive plasma molecules.

### 3.2. Virus Reduction by Exposure to CAP

Virus suspensions were tested by (RT-)qPCR immediately after CAP exposure, or 7 days after exposure, and compared to non-exposed controls. All CAP regimens effectuated an increase in PCR cycles required to obtain a positive fluorescence signal.

**Figure 2:**
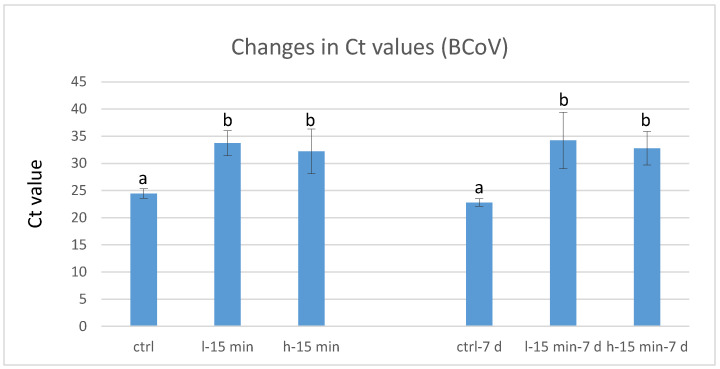
Ct values of BCoV samples exposed to CAP; ctrl…control (not exposed to CAP); l-15 min...15 min exposure to low-power CAP; h-15 min…15 min exposure to high-power CAP. **Left** columns show results immediately after CAP exposure; **right** columns show results after 7 days refrigerated storage; within left and right groups of columns, different letters indicate statistically significant differences of Ct values, *p* < 0.05; *n* = 6.

The experiments with BCoV showed strong reductions in viral loads. The samples stored for 7 days achieved an average increase of 11.4 (low power) or 10.0 (high power) Ct values (corresponding to −3.4 and −3.0 log), while the Ct values of the immediately frozen samples increased by 9.4 and 7.8 cycles, respectively, which corresponds to a reduction of −2.8 (low power) or −2.3 log cycles (high power) of the initial viral load (Figure 2). This reduction was statistically significant (*p* < 0.05) compared to the control.

**Figure 3:**
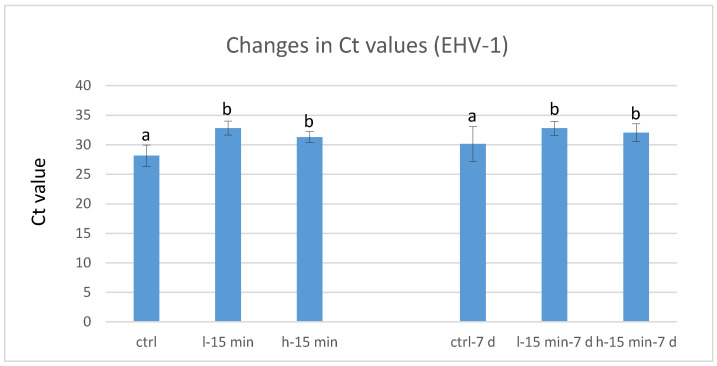
Ct values of EHV-1 samples exposed to CAP; ctrl…control (not exposed to CAP); l-15 min...15 min exposure to low-power CAP; h-15 min…15 min exposure to high-power CAP. **Left** columns show results immediately after CAP exposure; **right** columns show results after 7 days refrigerated storage; within left and right groups of columns, different letters indicate statistically significant differences of Ct values, *p* < 0.05; *n* = 6.

The increase in Ct values in EHV-1 samples exposed to CAP was less pronounced, yet statistically significant (*p* < 0.05). For immediately frozen samples, it was 4.7 Ct values (or −1.4 log) for “low power” and 3.2 Ct (−1.0 log) for “high power” (Figure 3). The Ct values of the samples stored for 7 days increased by 2.6 (−0.8 log) after “low power” treatment, and by 1.9 (−0.6 log) after “high power” exposure to CAP. Regardless of the virus isolate used, there was no significant difference between “low power” and “high power” treatment.

We used two culturable, virologically very different virus species as surrogates for food-contaminating viruses, i.e., one double-stranded DNA virus, EHV-1, and one single-stranded RNA virus, BCoV. We quantified the reduction in viruses by determining the Ct values before and after CAP exposure. In order to destroy any free DNA or RNA molecules that emerged during the treatment with CAP, and to ensure that only intact virus particles were detected, each EHV-1 sample was subjected to DNase treatment, and each BCoV sample to RNase treatment after exposure to CAP. The Ct value of (RT-)qPCRs represents a continuous, semi-quantitative measure providing information about the viral load in a sample. Numerous studies show a correlation between a low Ct value and clinical relevance. This includes the study by Fuller et al. [57], in which an association between the Ct value and the clinical severity of diseases could be demonstrated. Likewise, Wishaupt et al. [58] assessed the clinical relevance of the relationship between viral load, determined by the Ct value, and clinical disease in children with multiple viral respiratory infections. Thus, this value was used for the present study to demonstrate a possible reduction in the viral load by CAP. The reduction for the enveloped RNA virus was in the range of results reported for non-enveloped viruses (adenoviruses [7], hepatitis A virus [59]). The weak effect of CAP on the enveloped DNA virus would deserve additional studies on the action of plasma molecules on the envelopes of different viruses.

### 3.3. Changes in DMEM after CAP Exposure

Exposure to CAP at “low” and “high power” resulted in an increase in DMEM temperature within the first 5 min of exposure. While the temperature remained nearly stable with time under “low power”, a gradual increase was noted for the “high power” setting (Table 2). A similar trend was observed for the pH at “high power”, while at “low power” the temperatures after 5, 10, and 15 min did not differ. The ozone content in DMEM was determined immediately after exposure to CAP for 10 min, with 150–21 mg O_3_/L for “low power” and 90–150 mg O_3_/L for “high power” exposure (*n* = 3), whereas it was below the limit of detection in unexposed controls. Generally, ozone is considered a potent disinfectant with minimum or no effects on food items, since it decomposes rapidly [60] and leaves no residual ozone amounts; thus, no limits are established for food products.

The glucose content in DMEM decreased during CAP exposure from initially 4.72 ± 0.13 g/L to 4.50 ± 0.20 g/L at “low power” and to 4.40 ± 0.10 g/L at “high power” (*n* = 3), which corresponded to a statistically significant (*p* < 0.05) decrease of 4.7 and 6.8%, respectively. 

### 3.4. Changes in Tap Water and Salt Water after CAP Exposure

CAP exposure of tap water effectuated a drop in pH. After 5 min at “low power”, the pH dropped from 7.7 to 4.3. Then, the decrease slowed down, with a “final” pH (i.e., after 15 min CAP exposure) of 3.5. Similar results were obtained after “high power” exposure. A slightly delayed pH drop was noted in CAP-exposed salt water, with an initial pH of 8.0 ± 0.1 (Table 3). After 15 min at “low power”, the pH decreased by 5.19 and by 5.82 at “high power”. The temperate increased with time in both settings, by up to 7 °C after 15 min at “high power”.

Since the CAP reactive gas species have a rather limited depth of penetration (up to 1 μm [61]), liquid media were constantly agitated to allow better exposure of the liquid to the gas species. This was ensured by placing a 12-well plate with 600 µL per well at a shaker set to 800 rpm. Although CAP as a non-thermal antimicrobial treatment has been shown to have no, or only minimal, effects on quality traits of fresh produce and other susceptible food items [62,63,64], we encountered a different situation in liquid media.

Exposure of liquid media to CAP resulted in a temperature increase of up to 12 °C. We observed an only moderate pH increase of 0.2–0.6 units in the buffered, nutrient-rich DMEM, whereas in the unbuffered tap water and salt water, pH dropped by 0.6–5.8 units, most probably due to the dissolution of nitrate generated in the plasma. The slight pH increase in DMEM was expected in an atmosphere containing less than 10% CO_2_ (which is the case in ambient air) [65] and is not indicative of a CAP effect.

The reduction in glucose was however not expected, and should be explored in further studies. A potential explanation could be that exposure to CAP induces a Maillard reaction between the glucose and amino acids of DMEM. Similarly, CAP has previously been shown to induce a Maillard reaction when treating peanut protein [66,67].

Of particular relevance is the pH drop in salt water, the composition of which should allow holding of live oysters. This means that salt water would require a pH adjustment post-CAP in order to achieve the pH range of 7.1–8.2 necessary for oysters to survive [68]. This was of particular importance, since we wanted to test the applicability of cold plasma to sanitize salt water in holding tanks for oysters. We thus refrained from further experiments on liquids and studied oyster slurry.

### 3.5. Changes in Oyster Slurry after CAP Exposure

Rise in temperature was comparable to the findings for liquid media. This was not unexpected since our plasma source generated heat and no ventilation was provided, as ventilation might have blown away the plasma gas species. Appropriate measures to stabilize temperature should be considered if this treatment is to be used in commercial settings on temperature-sensitive food [62,63], e.g., by placing cooling units directly below the sample or by using a fan to direct the plasma cloud to the sample.

A decline in pH was observed in oyster slurry (Table 4), but less pronounced than in unbuffered liquid media (tap water and salt water). The CAP-exposed oyster samples did not show discolouration, which was confirmed by colour measurement before and after CAP exposure; this has also been reported by Choi et al. [39].

Since no statistical significance could be established for differences in L*, a*, and b* values between non-exposed and CAP-exposed oyster slurry (*n* = 3), we report only an average across all results, with L* = 41.1 ± 1.4; a* = 1.3 ± 0.7; b* = 17.6 ± 1.8; and a maximum ΔE* value of 2.7.

Nitrogen contents (initially 13.6 ± 0.1 g/kg oyster slurry) increased with increasing duration of CAP exposure, with a maximum increase of 6.1% after 15 min “low power” and of 12.4% after 15 min (“high power”). Since water loss due to evaporation was <2% in all settings, this increase was higher than could be expected due to evaporation. A similar increase, albeit at lower levels, was observed for nitrate-N, both under “low” and “high power” CAP exposure, whilst an increase in nitrite-N could be observed under “high power” CAP exposure only (Table 5). The increases in nitrite-N and nitrate-N combined are in the range of 30–50 mg N/kg, which is considerably lower than the increase in total N (~1200 mg N/kg). The generally higher N-levels under “high” compared to “low power” CAP exposure are in line with the dominance of N-compounds in plasma at higher energy levels [12].

In summary, we observed a significant increase in total N, but no significant changes in NO_2_ and NO_3_ contents. We expected that (in particular “high power”) CAP would effectuate an increase in NO_3_ in oyster slurry. Yet, we observed an increase in total N instead, so we assume that a fraction of the formed NO_x_ reacts with compounds of the oyster flesh (in particular haemocyanin), similar to the curing reaction observed in the flesh of mammals [43].

In order to corroborate our assumption of NO_x_ binding to oyster flesh, we mixed KNO_3_ with oyster flesh, corresponding to a gavage of 138 mg N/kg. In these samples, NO_3_ and NO_2_ contents in the flesh were determined at 15 min and 2 h (at room temperature, sealed) after mixing. Samples without addition of KNO_3_ served as controls. As shown in Table 6, the amounts of KNO_3_ recovered at 15 min and 24 h after nitrate addition were only approximately two thirds of the expected amount.

The observed increase in total N in the CAP-exposed oyster slurry may not be explained by the moderate weight loss (evaporation) of <2%. Most likely, this increase was attributable to dissolved NO_x_ from the plasma. Although we observed an increase in NO_3_ contents in oyster slurry, this could only in part explain the rise in total N, which led to the assumption that the dissolved NO_3_ reacts with oyster flesh. The loss of nitrate in oyster slurry mixed with KNO_3_ was in line with this assumption, and interestingly, also a rapid decline in nitrite contents was observed. This may have been accomplished by reducing activity in the oyster flesh, but it is unclear why, in the presence of such agents, high initial contents of nitrites and nitrates would be possible. Although this issue must be clarified in further studies, our data show that, irrespective of an unaltered appearance, oyster slurry was substantially changed in terms of higher nitrogen content, most likely due to a sort of curing. Admittedly, we did not record a shift in colour towards green (i.e., a decline in a* value [69]), as would be expected in “cured” haemocyanin [43]. While this would not necessarily qualify CAP-treated oyster flesh as a “novel food” according to EU legislation [52], it would require including information on the label and ultimately rendering CAP-treated unprocessed mussel (e.g., Ostreidae and Mytilidae) flesh as processed seafood. Likewise, we refrained from presenting the proof of concept, i.e., that the selected CAP regimen would inactivate viruses not only in DMEM, but also in an oyster-slurry matrix, since the mechanisms behind the observed change in nitrogen in oyster slurry need to be elucidated before attempting to establish the antiviral effect in this particular matrix.

We thus decided to clarify the nitrogen increase before studying the antiviral effect in an oyster-flesh matrix.

## 4. Conclusions

A cold atmospheric plasma generating device based on dielectric barrier discharge was highly effective in inactivating an enveloped RNA virus. Reactions of the plasma gas species proved to be the limiting factor when the CAP treatment was applied to salt water (oyster holding medium) or to oyster slurry. In both settings, reactive nitrogen species from the plasma cloud accumulated in the matrices, effectuating a drop of pH in salt water, and an increase in total nitrogen in oyster slurry.

With respect to live oysters, CAP could be used to sanitize the holding water; however, obviously, the plasma-induced pH drop has to be corrected before the water can be used in a holding tank. This might be accomplished in a circulating system, with CAP treatment outside of the holding tank, followed by pH adjustment and then redirecting the sanitized water back to the tank. In this setting, in-shell oysters would be sanitized indirectly by sanitizing the circulating holding water.

Analyses of nitrite and nitrate levels in the oyster slurry indicated that these compounds react with the oyster tissues, which raises concerns of whether such treated oysters would be either “processed” or “novel” foods according to EU legislation. We assume that this reaction involves in particular the haemocyanin compound. Further studies are suggested to explore the effects of nitrite on live oysters.

## Figures and Tables

**Table 1:** Settings of the DBD system. The settings for “high power” allow an increased production of nitrogen compounds such as nitrite and nitrate, while the “low power” variant creates ozone-dominated compounds.

Power	Power Input (P_in_) [W]	Output Voltage (V_out_) [kV]	Dissipated Power (P_out_) [W]	Power Density [W/cm^2^]
Low	20.70	8.16	17.87	0.48
High	29.90	9.44	25.38	0.67

**Table 2:** pH and temperature changes in DMEM at different times of exposure to CAP (*n* = 12).

Minutes	Temperature Increase in °C		pH Increase	
	Low Power	High Power	Low Power	High Power
0 to 5	8.6 ± 1.8	8.5 ± 3.1 ^a^	0.36 ± 0.27	0.24 ± 0.29 ^a^
0 to 10	8.8 ± 1.8	11.4 ± 2.1 ^b^	0.34 ± 0.31	0.46 ± 0.35 ^b^
0 to 15	8.7 ± 2.3	11.9 ± 2.1 ^b^	0.29 ± 0.52	0.57 ± 0.36 ^b^

Within columns, data with different superscript letters differ significantly, *p* < 0.05.

**Table 3:** pH and temperature changes in salt water at different times of exposure to CAP (*n* = 4).

Minutes	Temperature Increase in °C		pH Decrease	
	Low Power	High Power	Low Power	High Power
0 to 5	2.8 ± 1.2 ^a^	4.2 ± 1.1 ^a^	0.92 ± 0.08 ^a^	0.59 ± 0.28 ^a^
0 to 10	4.4 ± 0.3 ^b^	3.9 ± 0.9 ^a^	1.28 ± 0.02 ^a^	4.67 ± 0.81 ^b^
0 to 15	6.2 ± 0.2 ^b^	7.0 ± 2.3 ^b^	5.19 ± 0.55 ^b^	5.82 ± 1.87 ^b^

Within columns, data with different superscript letters differ significantly, *p* < 0.05.

**Table 4:** Changes in oyster slurry at different times of exposure to CAP (*n* = 6).

Minutes	Temperature Increase in °C		pH Decrease	
	Low Power	High Power	Low Power	High Power
0 to 5	6.5 ± 2.2 ^a^	9.2 ± 3.1 ^a^	0.09 ± 0.03 ^a^	0.16 ± 0.02 ^a^
0 to 10	8.1 ± 2.8 ^a,b^	11.0 ± 2.8 ^a,b^	0.11 ± 0.06 ^a^	0.17 ± 0.03 ^a^
0 to 15	9.2 ± 2.3 ^b^	13.6 ± 2.9 ^b^	0.24 ± 0.02 ^b^	0.21 ± 0.02 ^b^

Within columns, data with different superscript letters differ significantly, *p* < 0.05.

**Table 5:** N-content of oyster slurry exposed to CAP for different periods of time (5, 10, 15 min) at two energy levels (high, low), (*n* = 3).

mg/kg Fresh Matter	N (Kjeldahl Method)	N from Nitrate	N from Nitrite
Controls	13,520 ± 679 ^a^	705 ± 37 ^a^	297 ± 23 ^a^
**Low power**			
5 min	13,670 ± 1273 ^a^	724 ± 24 ^a^	304 ± 36 ^a^
10 min	14,015 ± 1768 ^b^	872 ± 16 ^b^	297 ± 23 ^a^
15 min	14,130 ± 424 ^b^	1019 ± 9 ^c^	308 ± 14 ^a^
**High power**			
5 min	14,225 ± 212 ^c^	764 ± 25 ^d^	284 ± 20 ^a^
10 min	14,210 ± 424 ^c^	1020 ± 19 ^e^	309 ± 39 ^a^
15 min	14,765 ± 248 ^d^	1095 ± 36 ^e^	332 ± 21 ^b^

Within columns, data with different superscript letters differ significantly, *p* < 0.05.

**Table 6:** Contents of nitrate and nitrite in oyster slurry (in mg/kg fresh matter), (*n* = 3).

Sample	Expressed as KNO_3_	Expressed as NaNO_2_	Expressed as N from KNO_3_	Expressed as N from NaNO_2_
Controls	441.0 ± 44.2	1293.0 ± 20.0	61.0 ± 6.1	262.0 ± 4.1
KNO_3_ addition (calculated)	1000.0	-	138.6	-
Total NO_3_ expected	1441.0	-	199.6	-
15 min after KNO_3_ addition	973.0 ± 3.6	37.0 ± 17.7	135.0 ± 0.5	7.0 ± 3.6
24 h after KNO_3_ addition	1097.0 ± 28.0	Not detected	152.0 ± 3.9	Not detected

## Data Availability

Datasets analysed in this study are publicly available. These data can be found here: https://phaidra.vetmeduni.ac.at (accessed on 26 November 2021).
